# A nonsense variant in *FBN1* caused autosomal dominant Marfan syndrome in a Chinese family: a case report

**DOI:** 10.1186/s12881-020-01148-1

**Published:** 2020-10-21

**Authors:** Yuping Niu, Sexin Huang, Zeyu Wang, Peiwen Xu, Lijuan Wang, Jie Li, Ming Gao, Xuan Gao, Yuan Gao

**Affiliations:** 1 Center for Reproductive Medicine, Cheeloo College of Medicine, Shandong University, Jinan, 250012 China; 2 National Research Center for Assisted Reproductive Technology and Reproductive Genetics, Shandong University, Jinan, 250012 China; 3 Key laboratory of Reproductive Endocrinology of Ministry of Education, Shandong University, Jinan, 250012 China; 4 Georgia Institute of Technology, Atlanta, GA USA

**Keywords:** Marfan syndrome, *FBN1* gene, Nonsense variant, Nonsense-mediated mRNA decay

## Abstract

**Background:**

Marfan syndrome (MFS) is a common autosomal dominant inherited disease, and the occurrence rate is around 0.1–0.2‰. The causative variant of *FNB1* gene accounts for approximately 70–80% of all MFS cases. In this study, we found a heterozygous c.3217G > T (p.Glu1073*) nonsense variant in the *FBN1* gene. This finding extended the variant spectrum of the *FBN1* gene and will provide a solution for patients to bear healthy offspring by preimplantation genetic testing or prenatal diagnosis.

**Case presentation:**

The patient was treated due to tachycardia during excitement in a hospital. Echocardiography showed dilatation of the ascending aorta and main pulmonary artery, mitral regurgitation (mild), tricuspid regurgitation (mild), and abnormal left ventricular filling. Electrocardiograph showed sinus rhythm. In addition, flutters of shadows in front of his eyes and vitreous opacity were present in the patient. Genomic DNA was extracted from peripheral blood samples from members of the family and 100 unrelated controls. Potential variants were screened out by next-generation sequencing and confirmed by MLPA & Sanger sequencing. Real-time fluorescence quantitative PCR (RT-qPCR) was performed to detect the relative mRNA quantitation in the patient. A heterozygous nonsense variant c.3217G > T of the *FBN1* gene, which resulted in p. Glu1073Term, was identified in both patients. Only wild type bases were found in the cDNA sequence of the patient. Real-time fluorogenic quantitative PCR results showed that the relative expression level of *FBN1* cDNA in the patient was only about 21% compared to that of normal individuals. This variant c.3217G > T of the *FBN1* gene introduces a Stop codon in the cb-EGF12 domain. We speculated that a premature translational-termination codon (PTC) was located in the mRNA and the target mRNA was disintegrated through a process known as nonsense-mediated mRNA decay (NMD), which led to a significant decrease of the fibrillin-1 protein, eventually causing clinical symptoms in the patient.

**Conclusions:**

In this study, we found a heterozygous c.3217G > T (p.Glu1073*) nonsense variant in the *FBN1* gene, which eventually led to Marfan syndrome in a Chinese family.

## Background

Marfan syndrome, or MFS, is a common autosomal dominant inherited connective tissue disorder with significant genetic diversity and variety in clinics. The incidence rate of MFS is around 0.1–0.2‰ [1] and about 0.1‰ in China [2]. MFS mainly devastates the skeletal, cardiovascular, and ocular systems, but all connective tissues around the body could potentially be affected [3]. MFS is primarily caused by a heterozygous variant of the *FBN1* gene located in 15q21.1. Currently, according to the HGMD database, over 2200 different pathological variants have been reported. The *FBN1* gene is composed of 65 exons that code the protein fibrillin 1, a vital component of connective tissues [4].

The *FBN1* gene encodes fibrillin-1. Fibrillin-1 is a 350kD calcium binding glycoprotein with modular structure and forty seven EGF (epidermal-growth-factor) structural domains of which forty three are cb-EGF (calcium binding) domains [5]. Each EGF site has six highly conserved cysteine residues, which forms disulfide bonds to maintain the 3D protein structure [6]. Six fibrillin-1 monomers combine to form a large complicated extracellular aggregate known as a “microfiber”, which is crucial to the integrity and stability of both elastic and inelastic tissues [4, 7]. A decrease in the number of microfibers or the abrupt loss of microfiber function would lead to interruption of the TGF-β signal [8], subsequently shutting down the pathway of fibrillin-1, largely disturbing the role of the skeletal, cardiovascular, and ocular systems.

In this study, 22 MFS relative genes were screened by next-generation sequencing so as to seek out the target variant in a MFS male patient, and a heterozygous c.3217G > T (p.Glu1073*) nonsense variant of the *FBN1* gene was discovered. This variant resulted in the relative lower expression level of *FBN1* in the patient than normal, which probably caused his MFS.

## Case presentation

### Patients and clinical presentation

Following the tenets of the Declaration of Helsinki, an informed consent was signed for the study and the experimental protocol was approved by the Ethics Committee. On the premise of informed consent, the peripheral blood of the pedigree members was collected for testing, and the peripheral blood of 100 phenotypically normal people was collected as a control [from volunteers with a normal phenotype in our center].

**Fig. 1:**
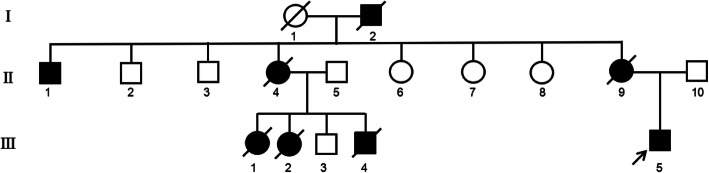
Pedigree chart of the patients

**Fig. 2:**
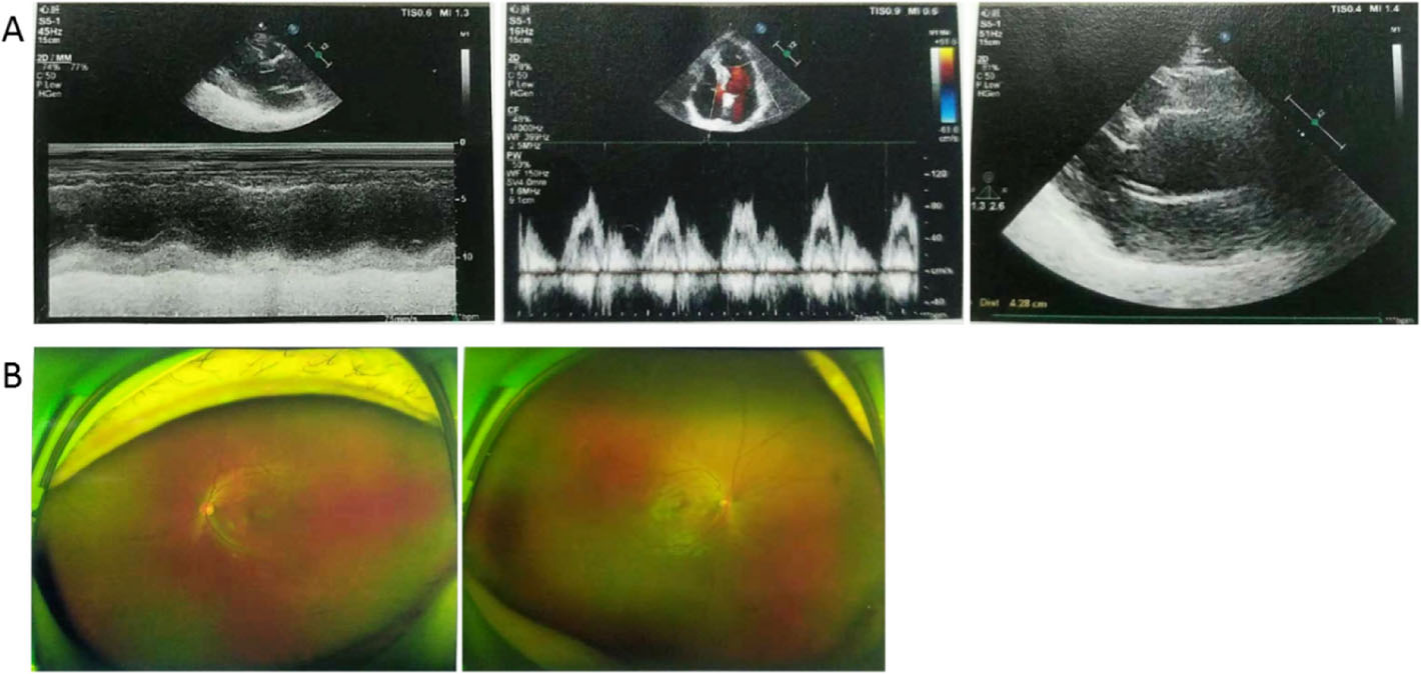
Clinical diagnosis of the proband. 2A: Ultrasonic cardiogram; 2B: Ocular ultrasound. Echocardiography showed dilatation of ascending aorta and main pulmonary artery, mitral regurgitation (mild), tricuspid regurgitation (mild), and abnormal left ventricular filling (**a**). Ocular ultrasound showed vitreous opacity (**b**)

An MFS family with three individuals in this study was genetically tested in Fig. 1 (II9, II10, III5). The patient (III5, Fig. 1) was an adult and was diagnosed as MFS with dilatation of the ascending aorta and main pulmonary artery, mitral regurgitation (mild), tricuspid regurgitation (mild), and abnormal left ventricular filling by echocardiography (Fig. 2a). Meanwhile, electrocardiograph showed sinus rhythm, and vitreous opacity was present (Fig. 2b). The fingers and toes of the patient’s mother (II9, Fig. 1) and aunt (II4, Fig. 1) had deformities, though his father did not have a specific abnormal phenotype. The patient’s grandfather (I2, Fig. 1) and uncle (II1, Fig. 1) are both highly myopic, and his grandfather (I2, Fig. 1) and three cousins (III1, III2, III4, Fig. 1) died of heart disease (they are all maternal relatives). In July 2018, the patient underwent molecular genetic testing in our center.

### Genetic analysis

Genomic DNA was extracted from peripheral blood samples from members of the family and 100 unrelated phenotypic normal controls using the Lab-Aid® 820 Nucleic Acid Extraction Mini Kit (Zeesan Biotech Co., Ltd., China). Twenty-two MFS relative genes were screened by next-generation sequencing for variant seeking (NovaSeq 6000, Illumina, USA), and large fragment deletions or repeated variants of the *FBN1* gene were detected by multiplex ligation-dependent probe amplification (MLPA) following the protocol of the SALSA MLPA P065 Marfan Syndrome-1 probemix and P066 Marfan Syndrome-2 probemix kit (MRC-Holland).

**Fig. 3:**
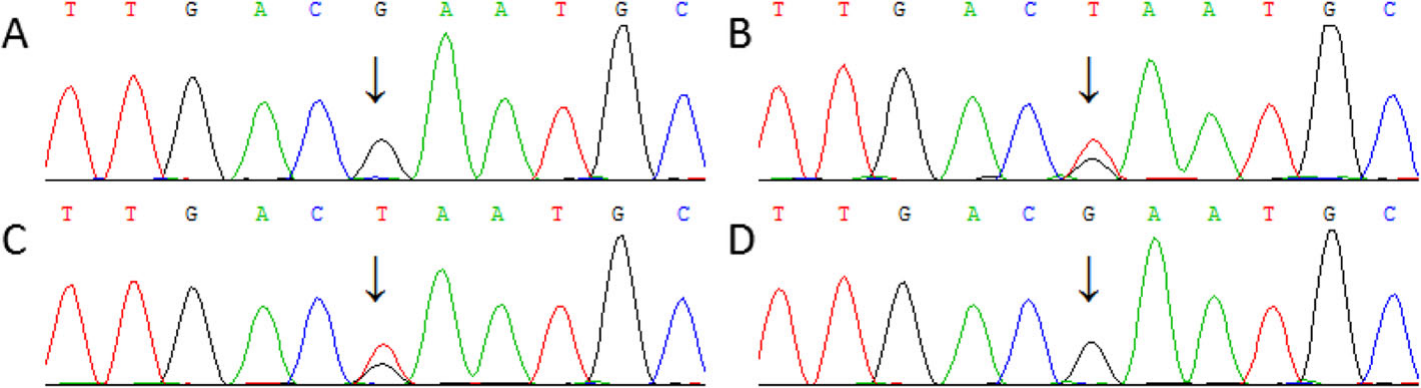
Genomic Sanger sequencing results of the patient, parents, and controls. **a** control group; **b**: the patient; **c** the patient’s mother; **d** the patient’s father. The arrow indicates the c.3217 position of the *FBN1* gene

**Fig. 4:**
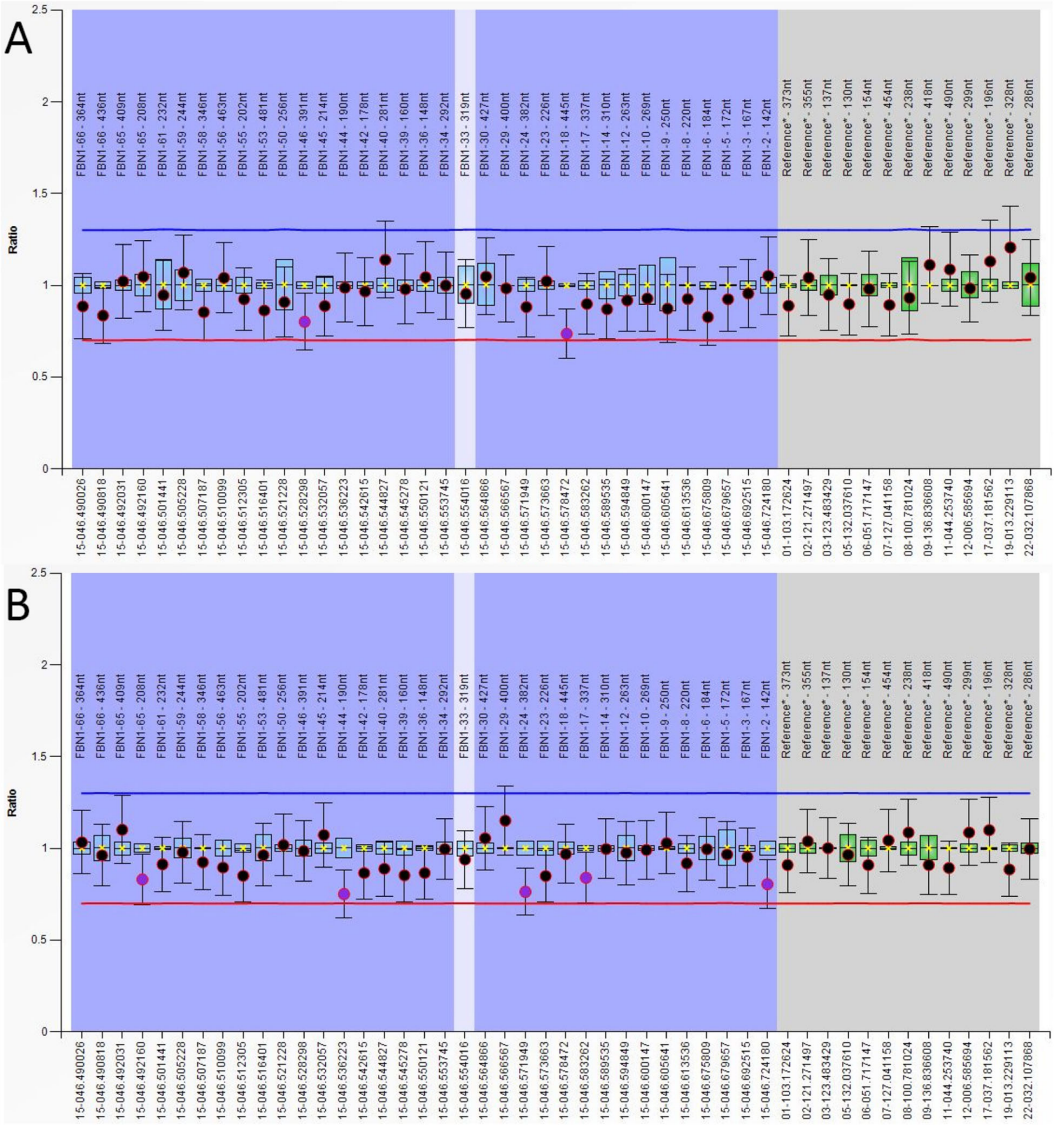
MLPA testing results of the patient. **a** MLPA P065 Marfan Syndrome-1 probemix; **b** MLPA P066 Marfan Syndrome-2 probemix

The candidate variants were confirmed and determined whether they co-segregated in this pedigree by Sanger sequencing. Primers were designed using Primer3 online software (http://bioinfo.ut.ee/primer3/) and synthesized by Invitrogen Biotechnology Co., Ltd. The target variants PCR products were sequenced by Sanger sequencing (BigDye® Terminator Cycle Sequencing Kit, ABI 3730, America). Sequencing data was blasted online (https://blast.ncbi.nlm.nih.gov/). One hundred unrelated control subjects were sequenced in the same way. Next-generation sequencing and Sanger sequencing data (Fig. 3) indicated a c.3217G > T heterozygous variant in the proband and his mother’s *FBN1* gene (NM_000138.4) but not in the gene of the father and 100 unrelated controls. MLPA results showed that no fragment loss or gain was found on the *FBN1* gene exons (Fig. 4).

**Fig. 5:**
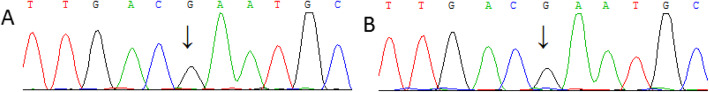
cDNA Sanger Sequencing results of the patient and the control. **a** the control; **b** The patient. The arrow indicates the c.3217 position of the *FBN1* gene, only wild G base was found at the position in both the patient and control

Total RNA was extracted from the blood samples using Trizol (Takara Dalian, China), and cDNA synthesis was performed using the PrimeScript™RT Master Mix (Perfect Real Time) (Takara Dalian, China). PCR and sequencing were performed in the same way as described above. cDNA Sanger sequencing results indicated that only the wild type G base was detected at the c.3217 position of the proband’s *FBN1* gene, and no T (variant) was found (Fig. 5).

**Fig. 6:**
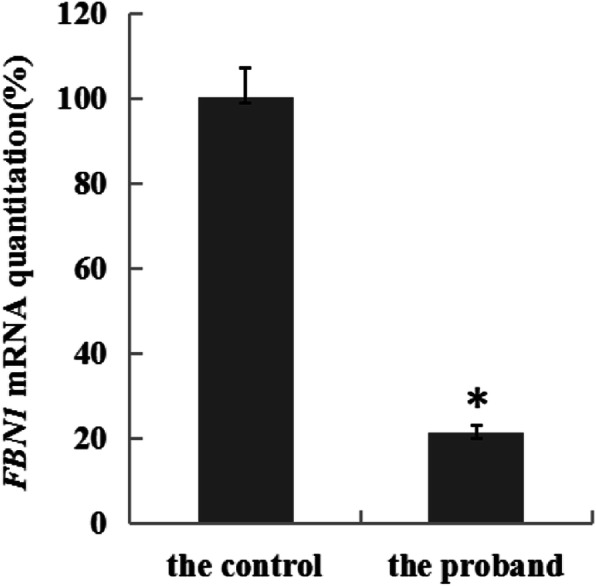
Relative expression level of *FBN1* gene of the proband is only about 21% compared to the control group. *β-actin* was a reference gene for analyses. The data represented the means of three independent experiments done in duplicates. *P*-value was assessed using a T-test. Significance *p* < 0.05 is indicated by (*); *p < 0.05 vs. control

Real-time fluorescent quantitative PCR was performed by the LightCycler 480 SYBR Green I Master Kit (Roche, Germany). The *β-actin* gene was used as a reference gene. Detection data were analyzed using the 2^-ΔΔCt^ method. The ratios of gene expression values were normalized using that of *β-actin*. A melting curve analysis was used to validate the reactions. The relative mRNA expression of the *FBN1* gene was normalized to the level of *β-actin* mRNA. All statistical data were compiled from ≥3 independent experiments. The following algorithm was used: ΔCt = Ct_FBN1_-Ct_β-actin_, ΔΔCt = ΔCt _the proband_ -ΔCt _the control_. The difference is statistically different when 0.01 < *P* < 0.05, and extremely notable when *P* ≤ 0.01. The melting curve of real-time fluorogenic quantitative PCR reflected a high specificity in both products of *FBN1* and *β-actin.* Compared to control groups, the relative expression level of the proband *FBN1* gene was only 21%, which was significantly lower than normal (*P* ≤ 0.05) and also much lower than heterozygous prospective expression (Fig. 6).

The variation classification of this variant was analyzed according to the American College of Medical Genetics and Genomics (ACMG) standards and guidelines [9]. The heterozygous c.3217G > T variant discovered in this study has not been documented in the HGMD database and is not reported in papers. Although this variant has been reported in ClinVar with accession VCV000803093 and has been reported in dbSNP with rs137854478, this variant was also found in neither the Exome Aggregation Consortium nor the 1000 Genomes Project population database. According to an OMIM report, the patient’s phenotype highly agrees with the symptoms of an autosomal dominant disorder known as Marfan syndrome caused by variants on the *FBN1* gene located on 15q21.1. This variation was supposed to disrupt the role of *FBN1* by a named NMD (nonsense-mediated mRNA decay) mechanism, which reduces gene production and affects protein function. We classified the variant as“pathogenic” (PVS1 + PM2 + PP4) according to the American College of Medical Genetics and Genomics (ACMG) standards and guidelines.

## Discussion and conclusions

Marfan syndrome is a hereditary connective tissue disease with high variability. Amongst over thirty different symptoms, the most prominent ones are lesions on the skeletal, cardiovascular, and ocular tissues [3]. In clinical cases, ectasia of aorta and aortic dissection are the two most lethal and important phenomena of MFS [10]. MFS caused by the FNB1 variant accounts for approximately 70–80% of all cases [11], while other cases may be caused by variants in potentially relative genes such as *ACTA2*, *MYH11*, *MYLK*, *SMAD3*, *TGFBR1*, *TGFBR2*, etc.

The *FBN1* gene, located on chromosome 15, q21.1, encodes 2871 amino acids. Currently, over 2200 different variations have been discovered, and most of them relating to MFS. There are two major types of influences that *FBN1* genetic variants can have on the structure of fibrillin-1: (1) nonsense variants, creating truncated proteins; (2) missense variants, leading to abnormal fibrillin-1 with mistakenly displaced amino acids [12]. In some nonsense variants, mRNA containing premature translational-termination codon (PTC) rarely generates truncated proteins because they are disintegrated through a process known as nonsense-mediated mRNA decay (NMD) [13]. In cases of missense variants, many displacements of amino acids influence components of crucial residues, such as the cysteine residue on cb-EGF structural domains. These displacements lead to possible mis-folding of structural domains and cause retention of the molecule in particular cases [14, 15]. Some other variants may affect the binding process between calcium and the cb-EGF domain, lowering the calcium affinity levels of the binding domain, altering the interaction patterns between cb-EGF domains in series with each other, and subsequently changing the rigidity of the molecule [16, 17].

In this research, we discovered that while the patient showed symptoms of classical MFS, a c.3217G > T heterozygous variant occurred on the26th exon of the *FBN1* gene. This nonsense variant turned the 1073th amino acid, a Glutamic acid, into a stop codon. In the meantime, only a wild type base was found in the cDNA sequence of the patients and the target mRNA expression level was only 21% compared to the controls. Thus, we speculated that the cell degraded mRNA through NMD, which led to a significant decrease in the expression level of the fibrillin-1 protein, eventually causing MFS [18, 19]. Tjeldhorn [20] et al. pointed out back in 1995 that nonsense or missense variants could cause alterations of mRNA expression, which agreed with our results. The mRNA relative expression level of another MFS patient (*FBN1* c.4414 T > C: p.Cys1472Arg) in our center tested by RT-PCR was 145.6% compared to the control samples, once again confirming the possible PTC mechanism.

On the other hand, a very similar variant of c.3217G > A, p.Glu1073Lys reported by Nijbroek [21], caused a severe symptom in newborn MFS. This variant affects the cb-EGD12 domain of the fibrillin-1 protein and each EGF-like domain has six highly conserved cysteine residues, which form disulfide bonds to establish the tertiary structure of the protein. The c.3217G > A: p.Glu1073Lys variant causes the replacement of glutamic acid by lysine, which changes the electric charge of the amino acid from negative to positive in the anterior of first cysteine in the domain. And it is highly correlated with a decrease in the binding affinity between the cb-EGF12 domain and calcium [22]. “Dominant negative mutation” is a term for a pathogenic mechanism currently proposed for many autosomal dominant diseases, including MFS [23, 24]. According to this model, a mutant gene product has the capacity to impair the function of the wide-type protein produced by the normal allele. When considering a multimeric protein, interaction between mutant monomer and the wild-type product can result in the formation of abnormal multimer and hence the disease phenotype [25]. This is why the variant of c. 3217 G > A caused a severe MFS symptom. The variant of c.3217G > T found in this study was a nonsense variant, which produced a premature translational-termination codon that reduces the stability of the variant transcript and consequently reduces protein production from the variant copy of the gene [26]. The production of the protein was decreased, but the protein produced by wild type gene was still there, giving rise to a mild MFS phenotype by haploinsufficiency. The mechanisms and pathway affected by different variants at the same DNA position would probably lead to entirely different phenotypes. This hypothesis requires further studies to be verified.

In conclusion, we discovered a nonsense variant of the *FBN1* gene, c.3217G > T, p.Glu1037Ter, which is very like to directly cause Marfan syndrome. This finding extended the variant spectrum of *FBN1* gene and will provide a solution for patients to bear healthy offspring by preimplantation genetic testing or prenatal diagnosis. This study is based on data at the molecular level, and more experiments are needed to clarify the mechanism in the future.

## Data Availability

All data generated or analyzed during this study are available from the corresponding author upon reasonable request. The RNA sequencing data has been uploaded to NCBI, and GenBank accession numbers are “BankIt2259986 Seq1 MN413940” and “BankIt2259986 Seq2 MN413941”.

## Abbreviations

MFS: Marfan syndrome
PTC: premature translational-termination codon
NMD: nonsense-mediated mRNA decay
